# A Mobile-Based Intervention to Increase Self-esteem in Students With Depressive Symptoms: Randomized Controlled Trial

**DOI:** 10.2196/26498

**Published:** 2021-07-12

**Authors:** Alina Bruhns, Thies Lüdtke, Steffen Moritz, Lara Bücker

**Affiliations:** 1 University Medical Center Hamburg-Eppendorf (UKE) Hamburg Germany

**Keywords:** mHealth, depression, depressive symptoms, students’ mental health, self-help smartphone app, mobile phone, self-esteem

## Abstract

**Background:**

Depressive symptoms are one of the most common and ever-increasing mental health problems among students worldwide. Conventional treatment options, particularly psychotherapy, do not reach all students in need of help. Internet- and mobile-based interventions are promising alternatives for narrowing the treatment gap.

**Objective:**

In the framework of a randomized controlled trial, we aim to investigate the effectiveness, acceptance, and side effects of a self-help smartphone app (*MCT & More*) based on cognitive behavioral therapy, mindfulness, acceptance and commitment therapy, and metacognitive training in a sample of students with self-reported depressive symptoms. Furthermore, we were interested in examining the influence of treatment expectations and attitudes toward internet- and mobile-based interventions on treatment adherence and effectiveness.

**Methods:**

A total of 400 students were recruited via open access websites and randomized to either the intervention group (n=200), who received access to the self-help smartphone app *MCT & More* for a period of 4 weeks, or to a wait-list control group (n=200). The Patient Health Questionnaire-9 (depression) served as the primary outcome parameter, and the Rosenberg Self-esteem Scale (self-esteem) and the global item of the World Health Organization Quality of Life-abbreviated version (quality of life) served as the secondary outcome parameters. The Attitudes Towards Psychological Online Interventions was used to measure attitudes toward internet- and mobile-based interventions. Outcome expectations were assessed using the Patient Questionnaire on Therapy Expectation and Evaluation, and side effects were assessed using the Inventory for Assessing Negative Effects of Psychotherapy.

**Results:**

Per-protocol (PP), complete-case, and intention-to-treat analyses showed a significantly higher reduction in depressive symptoms (PP: *F*_1,222_=3.98; *P*=.047; *d*=0.26) and a significantly higher increase in self-esteem (PP: *F*_1,220_=8.79; *P*=.003; *d*=0.40) in the intervention group than in the wait-list control group. Most participants regularly used the self-help smartphone app (91/120, 75.8%, at least once a week). The more positive the attitude toward internet- and mobile-based interventions (*r*=0.260; *P*=.004) and the more positive the outcome expectation (*r*=0.236; *P*=.009), the more frequently the self-help smartphone app was used.

**Conclusions:**

The effectiveness of the self-help smartphone app *MCT & More* was demonstrated among students with depressive symptoms compared with a wait-list control group. The app could be offered regularly as a low-threshold intervention to enhance students’ health.

**Trial Registration:**

German Clinical Trials Register DRKS00020941; https://tinyurl.com/pr84w6er

## Introduction

### Background

Universities worldwide are confronted with increasing rates of mental health problems among students [1]. In Germany, 15.6% of the university students state that they are currently affected by depressive symptoms (women: 16.9% and men: 14%) [2]. Adjustments to the new living environment (eg, moving away from home and new social environment), expectations of academic performance (eg, final grade in the master’s program), and other stressors resulting from the university program (50% of male and 65% of female students cite their studies as the most common cause of stress) render students particularly vulnerable to developing mental disorders [3]. Mental disorders such as depressive symptoms affect the social and general functioning of students, thereby negatively influencing the course of their study [4] and leading to a deterioration in academic performance. High depression scores among students have been shown to be associated with a low grade point average [5], and depressive symptoms during examination periods predict low future grades [6]. In addition, students with health problems (mental and physical) take long study durations, change their course of study or university more often, and are less likely to have a secure livelihood [4].

Despite their negative impact on functioning, mental disorders among students often remain undertreated [7]. The treatment gap can be attributed to the lack of available psychotherapy, especially in rural areas [8]; self-stigmatization [9]; fear of being stigmatized by others [10]; the preference to solve the problem independently [8]; fear of having to talk about one’s own problems to a psychotherapist; or the high treatment costs that may arise. Furthermore, depressive symptoms are often not recognized or misinterpreted by primary care physicians [11], leading to reduced help-seeking behavior [12]. Due to the treatment gap, universities are encouraged to initiate help offers that better reach the affected students [2].

### The Potential of Internet-Based Interventions in the Treatment of Depression

In Germany, virtually all individuals (>99%) aged between 16 and 44 years use the internet [13]. German students belong to the generation of *digital natives* (confident in using computer technology), so it is assumed that students can easily use internet-based interventions [14]. The benefits of internet-based interventions are, inter alia, the high level of autonomy and privacy. They can be used from any location and are often available free of charge or at a low cost. They are not intended to replace traditional psychotherapy but to expand conventional care [15]. In the last decade, numerous internet-based interventions, especially for the treatment of anxiety disorders and depression, have been developed and tested for their efficacy [16-18]. Systematic reviews and meta-analyses have shown that they can improve mental health problems such as depression, anxiety, and stress among students [14,19,20].

Internet-based interventions can be categorized as either guided or self-guided. Although guided internet-based interventions are supported by a therapist or a trained person (eg, via frequent email correspondence or telephone support), self-guided ones do not provide additional human support. A recent meta-analysis indicated that guided internet-based interventions show higher effect sizes than self-guided ones (guided: *g*=0.65 and unguided: *g=*0.27) [21]. However, self-guided internet-based interventions have the advantage that they can be made available to a broad population requiring few resources (no psychotherapists required, can be used at any time without waiting time, and low costs for users [22]), making self-guided internet-based interventions easier to implement at universities. Studies have shown that guided and unguided internet-based interventions show similar effectiveness in direct comparison (ie, when the same intervention is evaluated as guided and unguided) [23,24].

### Advantages of Mobile-Based Interventions

Smartphones are the most used technological devices among students on campus [25]. On average, students use their smartphones for approximately 5 hours a day and check their smartphones 28 times daily, which suggests that mobile-based interventions could be highly appealing to students. Mobile-based interventions have already proven to be an effective strategy for improving health-promoting behavior in the general population (eg, physical activity and weight control) [26]. Recent studies have also indicated the effectiveness of self-help smartphone apps in treating depressive symptoms in university students [27,28]. One of the benefits of mobile-based interventions is that smartphones can be accessed almost anytime and are independent of location [29,30]. Therefore, these exercises can be easily integrated into everyday life of students. Furthermore, mobile-based interventions provide the possibility to link users with other forms of support (eg, telephone numbers for acute crises) and to send reminders to the users. By sending reminders, the adherence of the users can be increased in self-guided treatment for anxiety and depression (eg, course completion with reminders: 58% and course completion without reminders: 35%) [31].

However, not all health care apps adequately protect the sensitive health data of the users (eg, commercialization of app users’ data) [32], and many German-language depression apps show limitations in quality (eg, in functionality, information quality, esthetics, and user involvement) [33]. Therefore, there is a need for high-quality apps, which are being investigated with regard to their benefits and risks [33]. Previous meta-analyses have found small effect sizes (Hedges *g*=0.22-0.33) [34,35] in reducing depressive symptoms. Furthermore, a recent study suggested that the actual treatment outcome (reduction in depressive symptoms) for mobile-based interventions can be predicted by the expected treatment outcome (usefulness for the patient: B=0.364 and perception of how logical the treatment is: B=0.528) [36].

### Objective of the Study

The overall goal of the study is to improve the health of students at German universities. The aim of this study is to examine the acceptance and efficacy of the self-help smartphone app *MCT & More* among German students with depressive symptoms in comparison with a wait-list control group. To our knowledge, little research has been conducted on the effectiveness and acceptance of self-help smartphone apps for students with depressive symptoms (especially in German-speaking countries).

A previous version of the app was positively evaluated by the users. In a randomized controlled trial (intervention group and wait-list control group) comprising 90 participants with reported depressive symptoms, it was shown that the app was effective in reducing depressive symptoms when used regularly (ie, several times a week, *P*=.05) app was effective in reducing depressive symptoms when used regularly (ie, several times a week) [37]. It was expected that the use of the self-help smartphone app would lead to a stronger reduction in depressive symptoms and to a higher increase in self-esteem and quality of life in the intervention group than in the wait-list control group after the intervention period. Another novel aspect we aimed to investigate was whether the effect of the app can be predicted by the attitudes toward the internet- and mobile-based interventions and the expected outcome. Moreover, we examined the possible side effects of self-help smartphone apps, which, to our knowledge, have barely been studied to date. Furthermore, an exploratory moderation analysis was conducted to identify possible moderators that affect differential symptom improvement (per-protocol [PP] sample).

## Methods

### Design

Two web-based assessments were performed at baseline (t0) and 4 weeks later (t1). All participants provided web-based informed consent at the beginning of the baseline assessment. No personal information was requested at any time, except for an anonymous email address (instructions to create an anonymous email address were given) and a personal codeword (consisting of the first letters of the parents’ names and some figures of their dates of birth). The collected data were anonymized and stored electronically on a password-protected computer. By providing the codeword or the anonymized email address, the data could be deleted at the request of the participants. At the end of the postassessment period, both groups were given access to a self-help manual, as an incentive, to improve emotional problems. As common in web-based trials, blinding of participants was not possible. The study was conducted in accordance with the Declaration of Helsinki. The local psychological ethics committee of the Center for Psychosocial Medicine of the University Medical Center Hamburg-Eppendorf assessed the study project as ethically unobjectionable (approval number: LPEK-0122).

### Participants

We recruited participants via web-based platforms and forums by posting an invitation to the study with a link to the web-based baseline assessment. At the beginning of the baseline assessment, participants received detailed information about the study’s goals and procedures and were informed about the underlying data protection. An electronic informed consent form was obtained from each participant.

The following inclusion criteria had to be met: student at a German university (whether the participants were actually students was checked by asking questions about the study system in Germany, which are difficult to answer correctly for nonstudents, eg, “What scoring system is used to measure your academic performance?”), aged at least 18 years, willing to provide informed consent, having access to the internet and a smartphone, having depressive symptoms (measured by Patient Health Questionnaire [PHQ-9], total score>0), willingness to participate in 2 pseudonymous web-based assessments, willingness to use the self-help smartphone app for a period of 4 weeks on one’s own responsibility, willingness to leave an anonymous email address, no acute suicidal tendencies (measured with item 9 of PHQ-9, cut-off>1), and no current or past bipolar or psychotic disorder. Other psychiatric diagnoses were not a criterion for exclusion. Parallel treatments (eg, psychotherapy or pharmacotherapy) could be continued during participation. If the inclusion criteria were not met, the participants were automatically excluded from the web-based assessment. Then, participants were informed about the reason for exclusion and received information about other help-seeking resources, such as telephone numbers for acute crisis.

**Figure 1:**
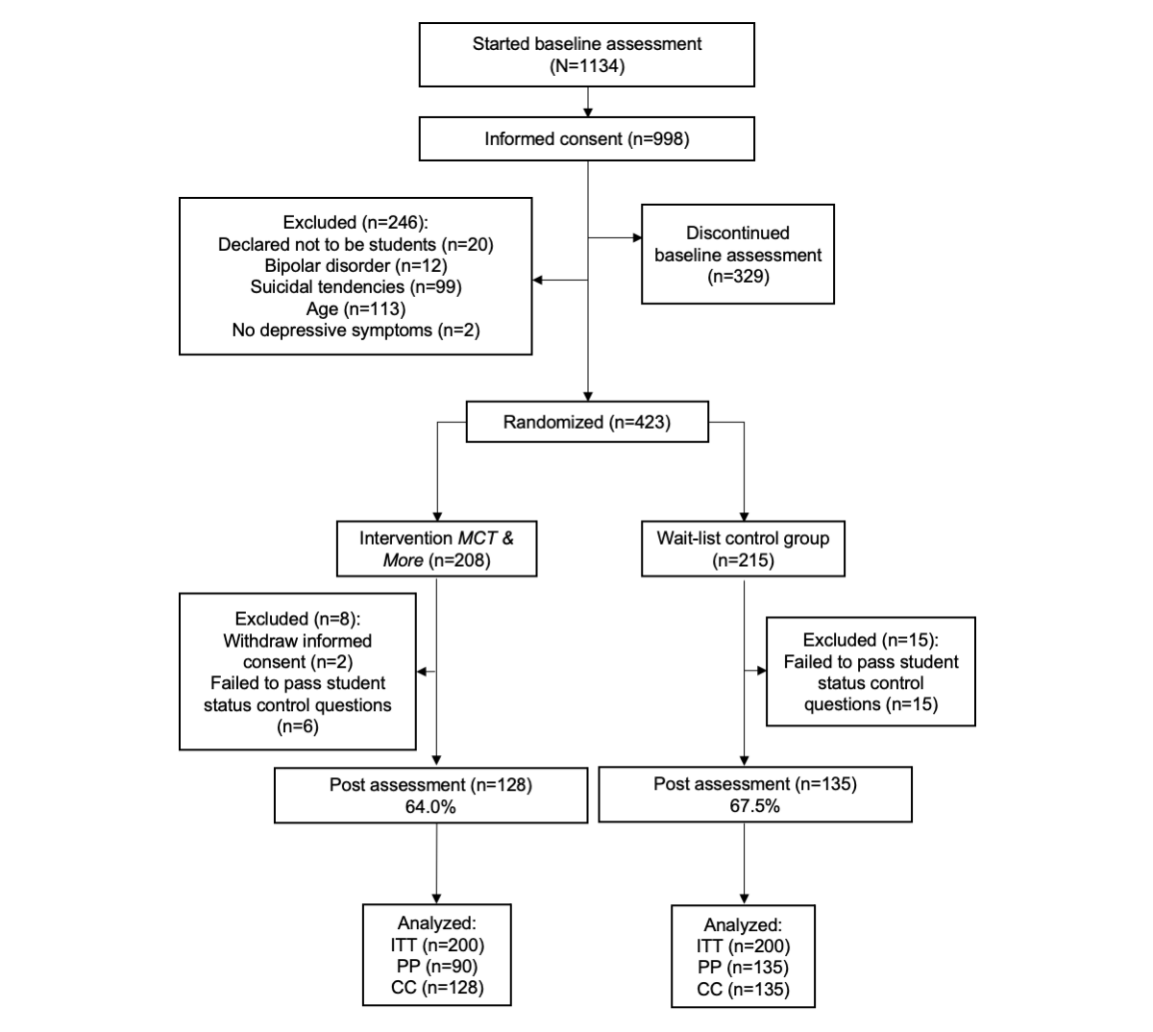
Flowchart. CC: complete-case; ITT: intention-to-treat; MCT: metacognitive training; PP: per-protocol.

Data collection took place in Germany from March 16, 2020 (first baseline assessment) to July 06, 2020 (last postassessment). During this period, Germany experienced the first wave of the COVID-19 pandemic. A total of 246 participants had to be excluded because the inclusion criteria were not met. The final sample consisted of 400 individuals (Figure 1).

### Procedure

At both measurement points (t0 and t1), data were collected using the survey software Qualtrics. Multiple registrations from one device were detected and prevented by the program. In the baseline assessment, sociodemographic and psychopathological data as well as the attitude toward internet- and mobile-based interventions and expected treatment outcomes were assessed. After the 4-week intervention period, all participants were invited via email to participate in the postassessment and were asked to provide their anonymous email address and personal code again to ensure a correct matching of predata and postdata. Afterward, the participants filled out the same psychopathological questionnaires used in the baseline assessment. In addition, the participants were asked about use frequency (“How often have you used the app during the last 4 weeks?”), side effects, and satisfaction with the self-help smartphone app (refer to the *Measures* section). The study was conducted at Hamburg-Eppendorf University Medical Center (Germany).

### Randomization

Randomization was performed using Qualtrics survey software after the baseline assessment. The option *equal distribution* ensured that there was a balanced distribution between the 2 groups. The allocation rule was set to 1:1.

### Sample Size

The calculation of the sample size for an analysis of covariance (ANCOVA) with 2 groups was performed using G*Power. The results indicated a sample size of 351 participants based on a small effect of *f*=0.15, with α=.05, and a power of 0.80. Considering a dropout rate of 15%, the final sample should include 413 participants. The calculation is based on the results of a meta-analysis investigating the effectiveness of smartphone app interventions for depression [34].

### Measures

#### PHQ-9: Depression Module

The self-assessment questionnaire PHQ-9 [38] is the depression module of the Patient Health Questionnaire and is used to measure symptoms of major depression according to the Diagnostic and Statistical Manual of Mental Disorders-IV. The symptoms are assessed using 9 items on a 4-point rating scale ranging from *not at all* (0) to *almost daily* (3). A total score between 0 and 27 can be calculated. Sum scores of 0-4 indicate none or minimal depressive symptoms, 5-9 indicate mild depressive symptoms, 10-14 indicate moderate depressive symptoms, and 15-27 indicate severe depressive symptoms. The internal consistency ranges from Cronbach α=.86 to .89 [38,39].

#### Rosenberg Self-esteem Scale

The Rosenberg Self-esteem Scale (RSE) is a self-assessment questionnaire presenting 10 statements on self-esteem, which are rated on a 4-point rating scale (1 to 4) from *strongly agree* to *strongly disagree*. The total score ranges from 10 to 40 points. High scores indicated high self-esteem. Its internal consistency ranges from Cronbach α=.77 to .88 [40].

#### World Health Organization Quality of Life-Abbreviated Version

In this study, the first item of the World Health Organization Quality of Life-abbreviated version (WHOQOL-BREF; “How would you assess your quality of life?”) with the response options *very poor* (1) to *very good* (5) was chosen to measure quality of life. The WHOQOL-BREF [41] is a self-assessment tool with 26 items (4 domains) and represents a short version of the questionnaire World Health Organization Quality of Life-100, which is based on the World Health Organization’s concept of quality of life. All domain scores were fairly to moderately correlated with the global quality of life [42,43]. In a sample of medical students, the internal consistency was Cronbach α=.896 [44].

#### Attitude Toward Psychological Online Interventions

Attitude Toward Psychological Online Interventions (APOI) [45] is a self-assessment tool consisting of 4 dimensions: (1) skepticism and risk perception, (2) trust in therapeutic efficacy, (3) perception of deficits in mechanization, and (4) perception of the advantages of anonymity. The questionnaire consists of 16 items and can be rated on a 5-point rating scale ranging from *do not agree at all* to *fully agree*. The total scale ranges from 16 to 80. A high total score indicates a positive attitude. All 4 dimensions were equally weighted. The APOI has an internal consistency of Cronbach α=.77 [45].

#### Patient Questionnaire on Therapy Expectation and Evaluation

The Patient Questionnaire on Therapy Expectation and Evaluation (PATHEV) [46] is a self-assessment questionnaire that measures therapy expectations and consists of 10 items. The instrument covers 3 subscales: (1) hope of improvement, (2) fear of change, and (3) suitability. Ten statements are presented, which are rated on a 5-point rating scale ranging from *not correct at all* to *completely correct*. The higher the sum of the subscales, the stronger the hope of improvement, fear of change, and suitability. The total scale ranges from 11 to 55, and the internal consistency ranges from Cronbach α=.73 to .83. The questionnaire was adapted to internet- and mobile-based interventions (eg, “I consider the treatment principle of psychological internet- and mobile-based interventions to be reasonable”).

#### Patient Satisfaction (Fragebogen zur Patientenzufriedenheit)

The instrument *Fragebogen zur Patientenzufriedenheit* (ZUF-8) [47] is the German version of the Client Satisfaction Questionnaire-8. The self-assessment questionnaire consists of 8 items that are used to assess patient satisfaction with a treatment, such as psychotherapy, in a 1D and global way. The items can be rated on a 4-point rating scale (eg, *excellent*, *good*, *less good*, and *bad*). A total score (8-32) can be calculated, whereby a high score indicates a high level of satisfaction. The internal consistency ranges from Cronbach α=.87 to .93 [47,48].

#### Inventory for Assessing Negative Effects of Psychotherapy

The Inventory for Assessing Negative Effects of Psychotherapy (INEP) [49] is a German self-assessment tool with 21 items and focuses on the side effects of psychotherapy regarding intrapersonal changes, partnership, stigma and financial worries, family, friends, dependency, and therapeutic relationship. The instrument consists of 2 scales: side effects (scale 1) and therapeutic misbehavior (scale 2). For the first 6 questions, a 7-point rating scale (−3 to +3, bipolar response format) can be used to indicate the extent to which the respective areas of life have developed positively or negatively from the start of the intervention or whether they have remained unchanged. A unipolar response format is used for questions 7-21 to determine whether a negative effect is experienced and with what intensity (0 to +3) it is perceived. A total score can be calculated for items 1-15, reflecting the number of experienced side effects. Furthermore, the intensity of the experienced side effects can be determined by calculating an average score (1-3, where 3 indicates a high intensity of the side effect). The INEP had an internal consistency of Cronbach α=.86 [49]. As no therapeutic relationship could be developed during the use of the self-help smartphone app, items 16-21 were excluded from the assessment. The wording was slightly adapted (self-help app instead of psychotherapy).

### Intervention

During the 4-week intervention period, the intervention group had free access to the self-help smartphone app *MCT & More* (Textbox 1), which is primarily intended for individuals with depressive symptoms.

**Figure 2:**
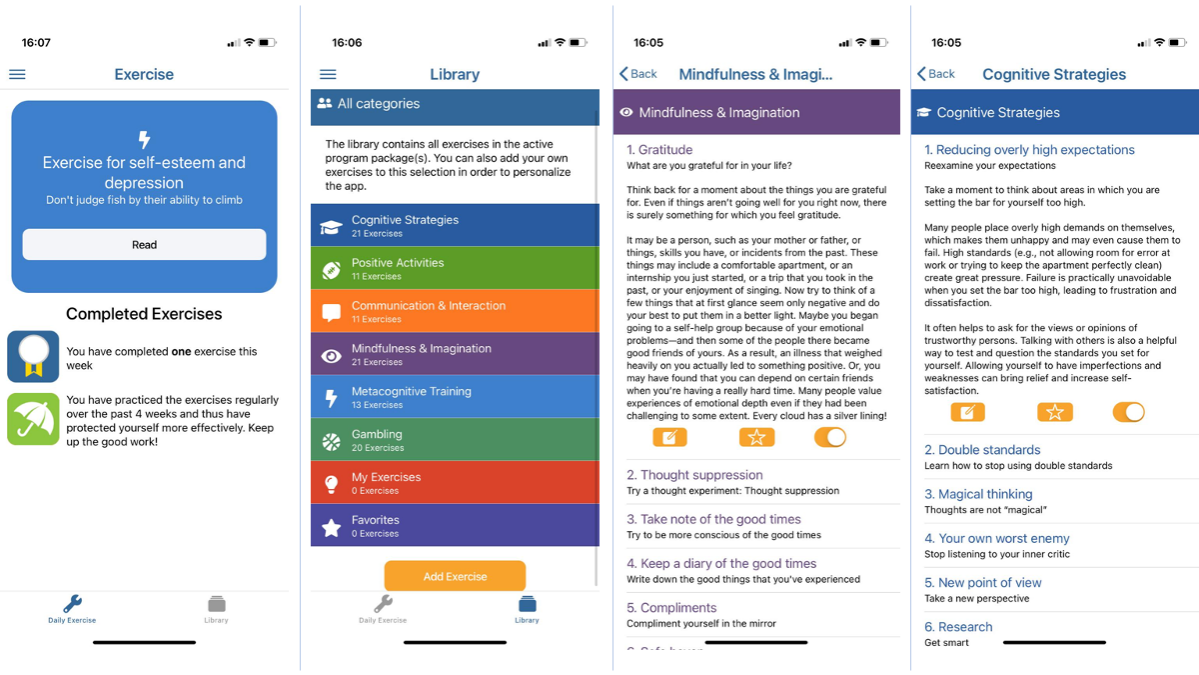
Screenshots of exercises.

The basic package of the self-help smartphone app *mood* comprises 57 short exercises on the following topics: cognitive strategies, communication and interaction, positive activities, and mindfulness and imagination. The program package *gambling* was developed especially for individuals with gambling problems, and the program package *metacognitive training* was intended for individuals with psychotic experiences. These program packages are deactivated by default settings but can also be useful for people who are not affected by the addressed symptoms. They can be activated in the app by the users themselves. The exercises take only a few minutes and are designed to be easy to use in the everyday life of students (Figure 2).

**Module descriptions of the MCT & More smartphone app.**

**Modules and Their Descriptions**

- Cognitive strategies
  Techniques to better perceive and understand own thinking and to break through adverse behavior patterns (eg, cognitive reframing and motivational techniques)
- Positive activities
  Behavior activation; interaction of body and emotion; and focus on positive things in life, own values and skills
- Communication and interaction
  Dealing with feedback, ways to improve communication skills, setting boundaries, and approaching others
- Mindfulness and imagination
  Conscious perception and experience of the current moment, acceptance, gratitude, focusing on positive things, and relaxation
- Metacognitive training
  Modification of cognitive biases and dysfunctional beliefs and reducing stigmatization and shame
- Gambling
  Modification of gambling-specific cognitive distortions, support with financial burdens, and dealing with relapses and gambling impulses
- My exercises
  Own exercises can be created

The contents and exercises are based on group metacognitive training (MCT) [50], cognitive behavioral therapy [51,52], and third wave techniques (eg, acceptance and mindfulness) [53,54]. The metacognitive training (MCT) was originally developed for people with psychosis [50]. Inspired by metacognitive training (MCT), a (group) training specifically for depression has evolved (metacognitive training for depression, D-MCT) [55]. Meta-analyses showed that metacognitive training (MCT) is effective in reducing anxiety, depression, and dysfunctional metacognitions (*g*=1.81-2.06) [56,57]. The app can be used as an add-on of metacognitive training for depression (D-MCT) but can also be used standalone. Metacognitive training for depression (D-MCT) focuses on the modification of cognitive biases and beliefs associated with the onset and maintenance of mental disorders such as psychosis and depression [50,55]. The training seeks to enable individuals to recognize and correct automatic and unconscious thought patterns. It also targets dysfunctional assumptions about thought processes as well as dysfunctional coping strategies (eg, social withdrawal, thought suppression, and rumination). On the basis of the principle that taking care of personal psychological well-being is a bit like brushing one’s teeth, the exercises should be performed regularly so that they become routine. Therefore, the app sends daily reminders via push messages. In addition, the *MCT & More* app contains gamification elements. Depending on the number of exercises completed, users can collect bronze, silver, or gold medals and obtain an open umbrella as a symbol for long-term protection. The app also offers the ability to create their own exercises. A learning algorithm, that is, an automatic adaptation to the user’s behavior, was not integrated (the app does not fall under the Medical Devices Act). The app *MCT & More* is currently available in German, English, Arabic, Turkish, Persian, and Serbian and can be downloaded free of charge for both Android and iOS operating systems.

The app has been continuously developed (eg, gamification elements, design, program packages *gambling* and *metacognitive training*, additional exercises in the other program packages, and various language versions) since the last evaluation [37]. The self-help smartphone app did not undergo major changes during the evaluation process of this study.

### Statistical Analyses

IBM Statistics 26 was used for statistical analysis. Independent samples *t* tests and chi-square tests were performed to compute group differences in baseline characteristics. Between-group differences over time (preintervention to postintervention) were calculated using ANCOVA with baseline scores as covariates. Pre-post differences were defined as within-group factors and groups as between-group factors. Paired samples *t* tests were used to analyze within-group differences. To determine the efficacy of the self-help smartphone app, intention-to-treat (ITT), PP, and complete-case (CC) analyses were performed. In the ITT analyses, all participants for whom baseline data were available were included in the evaluation. Missing data for the postvalues were calculated using expectation maximization. The PP analyses included only those participants who used the intervention as intended (at least once a week) and completed the postassessment. CC analyses included all participants who completed the postassessment (regardless of whether and how often the intervention was used). In the guidelines of the CONSORT (Consolidated Standards of Reporting Trials), it is recommended to perform both ITT and PP analyses in randomized controlled trials. With their conservative approach, ITT analyses comply with the guidelines of Good Clinical Practice and can be considered the gold standard for the evaluation of treatment effects [58,59]. In the PP analyses, the evaluation of the treatment effect is carried out under ideal conditions, so they provide an estimation of the actual efficacy. Furthermore, an explorative moderation analysis was carried out for the PP sample to identify possible moderators (included moderator variables were sociodemographic data, psychometric scales, and medication; Table 1) that affected differential symptom improvement (outcome measure: PHQ-9) using SPSS macro PROCESS by Hayes [60].

**Table 1:** Demographic description of the intention-to-treat sample (N=400).

Baseline characteristics				Intervention group (n=200)	Wait-list control group (n=200)	Chi-square test (*df*)	*t* test (*df*)	*P* value
**Sociodemographic data**								
	Male, n (%)			24 (12)	19 (9.5)	1.0 (2)	N/A^a^	.60
	Age (years), mean (SD)			23.13 (3.56)	22.84 (3.15)	N/A	−0.86 (398)	.39
	German, n (%)			190 (95)	194 (97)	1.0 (1)	N/A	.31
	School education (years), mean (SD)			12.39 (1)	12.48 (0.92)	N/A	0.91 (398)	.37
**Marital status, n (%)**						1.3 (3)	N/A	.73
	Single			99 (49.5)	94 (47)			
	Relationship			95 (47.5)	101 (50.5)			
	Married			5 (2.5)	5 (2.5)			
	Divorced			1 (0.5)	0 (0)			
**Field of study, n (%)**						5.1 (6)	N/A	.53
	Engineering			4 (2)	3 (1.5)			
	Natural sciences			9 (4.5)	13 (6.5)			
	Medical science or health			61 (30.5)	59 (29.5)			
	Legal sciences or economics			24 (12)	19 (9.5)			
	Linguistics or culture			10 (5)	6 (3)			
	Social sciences			88 (44)	90 (45)			
	Others			4 (2)	10 (5)			
Semester, mean (SD)				5.6 (3.63)	5.57 (3.57)	N/A	−1.18 (398)	.24
**Psychometric scales or psychiatric disorders, n (%)**								
	None			100 (50)	104 (52)	0.2 (1)	N/A	.69
	Anxiety			54 (27)	40 (20)	2.7 (1)	N/A	.10
	Depression			72 (36)	69 (34.5)	0.1 (1)	N/A	.75
	PTSD^b^			11 (5.5)	11 (5.5)	0.0 (1)	N/A	.99
	Alcohol or drug addiction			7 (3.5)	1 (0.5)	4.6 (1)	N/A	.03
	OCD^c^			5 (2.5)	17 (8.5)	6.9 (1)	N/A	.008
	Eating disorder			9 (4.5)	9 (4.5)	0.00 (1)	N/A	.99
	Personality disorder			9 (4.5)	1 (0.5)	6.6 (1)	N/A	.01
	ADD^d^			4 (2)	2 (1.0)	0.7 (1)	N/A	.41
	Others			5 (2.5)	2 (1)	0.4 (1)	N/A	.56
**Medication, n (%)**								
	None			155 (77.5)	157 (78.5)	0.1 (1)	N/A	.81
	Antidepressants			16 (8.0)	17 (8.5)	0.0 (1)	N/A	.86
**Measurements, mean (SD)**								
	PHQ-9^e^			11.13 (4.99)	10.98 (4.42)	N/A	−0.31 (398)	.76
	WHOQOL-BREF^f^			3.64 (0.85)	3.7 (0.75)	N/A	0.69 (398)	.49
	RSE^g^			25.73 (6.12)	26.62 (5.83)	N/A	1.49 (398)	.14
	APOI^h^			50.27 (7.34)	49.92 (8.06)	N/A	−.46 (398)	.65
	PATHEV^i^			35.69 (5.61)	36.35 (5.07)	N/A	1.15 (398)	.25
**Psychotherapy experiences**								
	**Previous treatments, n (%)**					5.3 (3)	N/A	.15
		None		107 (53.5)	109 (54.5)			
		Short-term		39 (19.5)	49 (24.5)			
		Long-term		27 (13.5)	28 (14)			
		More than one		27 (13.5)	14 (7)			
	**Assessment, n (%)**					10.7 (3)	N/A	.01
		Positive		72 (36)	59 (29.5)			
		Neutral		23 (11.5)	33 (16.5)			
		Negative		20 (10)	7 (3.5)			
	**Others**							
		**Fear of stigma, n (%)**				4.5 (3)	N/A	.21
			Yes or rather yes	127 (63.5)	121 (60.5)			
			No	73 (36.5)	79 (39.5)			

^a^N/A: not applicable.

^b^PTSD: posttraumatic stress disorder.

^c^OCD: obsessive-compulsive disorder.

^d^ADD: attention deficit disorder.

^e^PHQ-9: Patient Health Questionnaire-9.

^f^WHOQOL-BREF: World Health Organization Quality of Life-abbreviated version.

^g^RSE: Rosenberg Self-esteem Scale.

^h^APOI: Attitude Toward Psychological Online Interventions.

^i^PATHEV: Patient Questionnaire on Therapy Expectation and Evaluation.

## Results

### Sample Characteristics

A total of 400 participants (intervention group: 200 and wait-list control group: 200) were included in the analyses. Table 1 shows the demographic and psychopathological data of the participants at baseline.

The overall sample had an average age of 22.98 years (SD 3.36) and consisted of 10.8% (43/400) men and 88.5% (354/400) women. In addition, 0.8% (3/400) of participants stated diverse as their gender. The average PHQ-9 score was 11.1 (SD 4.71; moderate symptoms 10-14). Among the participants, 4.3% (17/400) met the criteria for severe depressive symptoms (PHQ-9 score>19), 20.3% (81/400) for moderately severe depressive symptoms (PHQ-9 score=15-19), 35.8% (143/400) for moderate depressive symptoms (PHQ-9 score=10-14), 32.3% (129/400) for mild depressive symptoms (PHQ-9 score=5-9), and 7.5% (30/400) for minimal depressive symptoms (PHQ-9 score=1-4). In addition, 46% (184/400) of participants stated that they had received psychotherapeutic treatment at least once.

The randomization was successful (Table 1). There were no significant differences between the groups in terms of age and gender or in primary and secondary outcome parameters (depressive symptoms, self-esteem, and quality of life). There were also no significant differences between the groups in terms of expected treatment outcomes and attitudes toward internet- and mobile-based interventions. However, the intervention group showed a significantly higher number of participants with an alcohol and drug addiction (intervention group: n=7; wait list control group: n=1) as well as a personality disorder (intervention group: n=9; wait list control group: n=1) and a significantly lower number of participants with an obsessive-compulsive disorder (intervention group: n=5; wait list control group: n=17). In addition, participants in the wait-list control group reported a neutral experience with psychotherapy more often (wait list control group: n=33; intervention group: n=23) and a positive experience (wait list control group: n=59; intervention group: n=72) and negative experience (wait list control group: n=7; intervention group: n=20) less often (Table 1).

### Within-Group Differences

The results of paired samples *t* tests showed a significant reduction in depressive symptoms, both in the intervention group (t_89_=4.88; *P*<.001; *d*=−0.38) and in the wait-list control group (t_134_=2.7; *P*=.007; *d*=−0.21) from t0 to t1 (Table 2).

Results of paired samples *t* tests also indicated a significant increase in scores on the self-esteem scale (RSE) for the intervention group (t_89_=−6.47; *P*<.001; *d*=0.38) and the wait-list control group (t_132_=−3.46; *P*=.001; *d*=0.16). For both groups, the results of the paired samples *t* test did not show a significant increase in quality of life (WHOQOL-BREF).

**Table 2:** Outcome measures at each assessment time for per-protocol sample (used program at least once a week; n=225).

Measurements	Intervention group (n=90)				Wait-list control group (n=135)			
Questionnaires	Pre, mean (SD)	Post, mean (SD)	Cohen *d* (95% CI)	*P* value	Pre, mean (SD)	Post, mean (SD)	Cohen *d* (95% CI)	*P* value
PHQ-9^a^	11.27 (5.03)	9.30 (5.22)^b^	−0.38 (−0.8 to 0.03)	<.001	11.10 (4.42)	10.17 (4.32)^c^	−0.21 (−0.55 to 0.13)	.007
RSE^b^	25.56 (6.41)	28.00 (6.44)^b^	0.38 (−0.04 to 0.80)	<.001	26.59 (6.02)	27.57 (6.43)^b^	0.16 (−0.18 to 0.50)	.001
WHOQOL-BREF^c^	3.74 (0.80)	3.86 (0.82)	0.15 (−0.27 to 0.65)	.12	3.67 (0.76)	3.76 (0.72)	0.12 (−0.22 to 0.46)	.19

^a^PHQ-9: Patient Health Questionnaire-9.

^b^RSE: Rosenberg Self-esteem Scale.

^c^WHOQOL-BREF: World Health Organization Quality of Life-abbreviated version.

### Between-Group Differences

For the primary outcome parameter (depressive symptoms, PHQ-9), the results of the ANCOVA were significant for ITT (*F*_1,398_=3.94; *P*=.048), PP (*F*_1,223_=3.98; *P*=.047), and CC (*F*_1,261_=4.60; *P*=.03; Table 3).

For reducing depressive symptoms, a small effect size of η_p_²=0.018 (*d*=0.26) was found in the PP sample. Furthermore, the results of the ANCOVA showed statistical significance for the secondary outcome parameter self-esteem (RSE) for ITT (*F*_1,398_=6.80; *P*=.009), PP (*F*_1,221_=8.79; *P*=.003), and CC (*F*_1,259_=7.26; *P*=.008). The analyses resulted in a small to medium effect size for the increase in self-esteem (η_p_²=0.038; *d*=0.40) in the PP sample across time. There was no significant improvement across time in quality of life (WHOQOL-BREF), as analyzed using an ANCOVA with baseline score as covariate in any of the samples: (ITT: *F*_1,398_=0.56; *P*=.46; PP: *F*_1,223_=0.41; *P*=.52; and CC: *F*_1,261_=0.81; *P*=.37).

**Table 3:** Analysis of covariances with respective baseline values as covariates.

Measurements	CC^a^ (n=263)			PP^b^ (n=225)				ITT^c^ (n=400)		
	*F* test (*df*)	*P* value	η_p_²	*F* test (*df*)	*P* value	η_p_²	*F* test (*df*)		*P* value	η_p_²
PHQ-9^d^	4.60 (1,398)	.03	0.017	3.98 (1,222)	.047	0.018	3.94 (1,261)		.048	0.010
RSE^e^	7.26 (1,398)	.008	0.027	8.79 (1,220)	.003	0.038	6.80 (1,259)		.009	0.017
WHOQOL-BREF^f^	0.81 (1,398)	.37	0.003	0.41 (1,223)	.52	0.002	0.56 (1,261)		.47	0.001

^a^CC: complete-cases.

^b^PP: per-protocol.

^c^ITT: intention-to-treat.

^d^PHQ-9: Patient Health Questionnaire-9.

^e^RSE: Rosenberg Self-esteem Scale.

^f^WHOQOL-BREF: World Health Organization Quality of Life-abbreviated version.

### Study Completion and App Use

Out of 400 participants, 263 (65.8%) completed the postassessment, 128 (64%) in the intervention group and 135 (67.5%) in the wait-list control group. Regarding study completion, there was no difference between the groups (*χ*²_1_=0.5; *P*=.46). Furthermore, participants who completed the study differed only in terms of their treatment expectations. Participants who completed the study expected a more positive treatment outcome (t0) than participants who did not complete the study (t_398_=−2.12; *P*=.04).

In the intervention group, 60% (120/400) of participants reported how often they used the self-help smartphone app during the intervention period (completed the daily exercise). The self-help smartphone app was used by 23.3% (28/400) of participants daily, by 17.5% (21/400) of participants 4-6 times a week, by 25% (30/400) of participants 2-3 times a week, by 10% (12/400) of participants once a week, by 19.2% (23/400) of participants 1-3 times in total, and by 5% (6/400) of participants not at all. The improvement in symptoms (PHQ-9) did not correlate with use frequency (*r*=0.020; *P*=.83). However, use frequency correlated with the expected treatment outcomes (*r*=0.236; *P*=.009) and attitude toward internet- and mobile-based interventions (*r*=0.260; *P*=.004; 1=not at all to 6=daily). The more positive the attitude toward internet- and mobile-based interventions and the more positive the expected treatment outcomes, the more often the self-help smartphone app was used.

### Attitude and Expectation

In total, of the 400 participants, 232 (58%) had a positive attitude toward internet- and mobile-based interventions, 30 (7.5%) had a neutral attitude toward internet- and mobile-based interventions, and 138 (34.5%) had a negative attitude toward internet- and mobile-based interventions. Although 87.3% (349/400) of participants believed that internet- and mobile-based interventions are therapeutically effective (optimism regarding personal therapeutic goal clarification, emotional expectation of helpful efficacy, expectation of learning new skills, cognitive acceptance of the methodology), 31.8% (127/400) of participants also stated that they were skeptical about internet- and mobile-based interventions and that they perceived risks (regarding professionalism, side effects, and feasibility). In addition, 67.8% (271/400) of participants perceived difficulties caused by automation (poor crisis management, low learning success, poorer cognitive understanding of therapy contents, and lower motivation because of lack of personal contact). The advantages of anonymity were reported by 45.5% (182/400) of participants (increased discretion, personally increased self-autonomy, reduction of self-stigmatization, and stigmatization by other persons).

Of the 400 participants, 257 (64.3%) indicated a positive expectation and 117 (29.3%) indicated a negative expectation of treatment outcome regarding the self-help smartphone app. Approximately half of the participants (191/400, 47.8%) did not expect the self-help smartphone app to reduce their symptoms and indicated that this is not the right program for them (172/400, 43%). Only a few participants (38/400, 9.5%) were afraid of change as a result of the self-help smartphone app. The effectiveness of the app could not be predicted by attitudes toward internet- and mobile-based interventions (β=−.006; t_275_=−0.17; *P*=.87; *R^2^*<0.001; *F*_1,275_=0.03; *P*=.87).

### Side Effects

Of the 119 participants (intervention group) who completed the questionnaire on side effects (INEP), 51 (42.9%) reported at least one positive side effect. The most commonly reported positive side effect was that participants felt better when using the self-help smartphone app (43/119, 36.1%). Furthermore, 17.6% (21/119) of participants stated that they experienced less pain from events from the past, 14.3% (17/119) stated that they experienced fewer conflicts in their partnership, 13.6% (16/119) stated that they had a better relationship with their friends, 10.9% (13/119) stated that they had a better relationship with their family, and 8.4% (10/119) stated that trusting others was easier for them.

Overall, of the 119 participants 27 (22.7%) reported a negative side effect. Fear of stigmatization was the most common negative side effect (12/119, 10.1%). In addition, 6.7% (8/119) of participants reported that they had longer phases in which they felt bad, 5.9% (7/119) of participants reported that they had problems with insurance, 5% (6/119) of participants reported that they experienced more pain from events from the past, 1.7% (2/119) of participants reported that they felt worse, 1.7% (2/119) of participants reported that they were more concerned about financial issues, 0.8% (1/119) of participants reported that trusting others is more difficult for them, 0.8% (1/119) of participants reported that they had a worse relationship with their family, and 0.8% (1/119) of participants reported that they had a worse relationship with their friends. None of the participants stated that they had changed as a person to the negative, that they had suicidal thoughts or intentions for the first time, or that they experienced more conflicts in their partnership.

The most intense change was observed in the improvement (positive side effect; mean 2.62, SD 0.65) and deterioration (negative side effect; mean 3.00, SD 0) of the relationship with the families. In comparison with the negative side effects, positive side effects were mentioned 3 times more frequently (negative: 41/158, 25.9% and positive: 120/158, 75.9%).

### Moderation Analysis

The results of the interaction effect of the explorative moderation analysis are shown in Table 4.

The analysis revealed that participants in the intervention group who had a higher expectation of treatment outcome (*P*=.02; PATHEV total score) and more hope (*P*=.049; PATHEV hope scale) showed a higher improvement in depressive symptoms (PHQ-9) than the wait-list control group. In addition, participants in the intervention group who were more worried that the app would not help them (*P*=.03) and participants in the intervention group who stated a higher reduced or excessive need to eat (*P*=.02) showed a less improved outcome (PHQ-9) than the wait-list control group.

**Table 4:** Moderators for Patient Health Questionnaire-9 improvement (dependent variable: Patient Health Questionnaire-9 total difference scores and independent variable: group, means are centered); results of per-protocol sample (N=225).

Moderator	B^a^ (SE)	*t* test (*df*)	*P* value	LLCI^b^	ULCI^c^	*P* value^d^ for −1 SD	*P* value^d^ for 0	*P* value^d^ for +1 SD
PATHEV^e^ total scale	0.218 (0.090)	2.428 (117)	.02	0.041	0.396	.65	.04	.002
PATHEV hope scale	0.321 (0.162)	1.982 (117)	.049	0.002	0.641	.90	.07	.006
PATHEV item 1	−1.023 (0.464)	−2.204 (117)	.03	−1.937	−0.108	.004	.14	.70
PHQ-9^f^ item 5	−1.058 (0.466)	−2.272 (223)	.02	−1.975	−0.140	.003	.01	.36

^a^B: interaction coefficient.

^b^LLCI: lower limit confidence interval.

^c^ULCI: upper limit confidence interval.

^d^The last 3 columns present the simple slopes.

^e^PATHEV: Patient Questionnaire on Therapy Expectation and Evaluation.

^f^PHQ-9: Patient Health Questionnaire-9.

### Subjective Appraisal

In the intervention group, 119 participants completed the questionnaire on patient satisfaction (ZUF-8). Table 5 shows the users’ subjective appraisal for each item.

The average total score was mean 20.28 (SD 5.36; 8=very dissatisfied to 32=very satisfied). The quality of the self-help smartphone app was rated positively by 64.7% (77/119) of participants. For each item, the positive evaluations outweighed the negative evaluations. In addition to ZUF-8, 3 further questions were asked regarding participant satisfaction. The majority of participants found the language 84.9% (101/119), text length 77.3% (92/119), and number of exercises 49.6% (59/119) in the self-help smartphone app to be just right.

**Table 5:** Subjective appraisal using *Fragebogen zur Patientenzufriedenheit* of *MCT & More* (n=119).

ZUF-8^a^ item	Mean (SD)	Positive^b^, n (%)
1. How do you rate the quality of the program? (excellent, good vs not that good, or not good)^c^	2.29 (0.61)	77 (64.7)
2. Did you receive the type of treatment you expected to receive? (absolutely, a lot vs a little, or not at all)	2.45 (0.78)	59 (49.6)
3. To what extent did the program help you cope with your problems? (absolutely, a lot vs a little, or not at all)^c^	2.63 (0.79)	56 (47.1)
4. Would you recommend the program to a friend with similar symptoms? (yes, probably yes vs probably not, or no)	2.61 (0.92)	52 (43.7)
5. How happy are you about the extent of the help you have received through using the program? (very satisfied, mostly satisfied vs somewhat dissatisfied, or dissatisfied)	2.50 (0.82)	66 (55.5)
6. Did the program help you to cope with your problems more successfully? (absolutely, a lot vs a little, or not at all)^c^	2.39 (0.63)	66 (55.5)
7. How satisfied are you with the program in general? (very satisfied, mostly satisfied vs somewhat unsatisfied, or unsatisfied)^c^	2.42 (0.85)	67 (56.3)
8. Would you use the program again? (Yes, probably yes vs probably not, or no)	2.44 (0.92)	59 (49.6)

^a^ZUF-8: Fragebogen zur Patientenzufriedenheit (German version of the Client Satisfaction Questionnaire-8).

^b^4-point rating scale: 1 and 2 were rated as negative and 3 and 4 as positive. In the table it is stated how often the question has been answered positively (rated 3 or 4).

^c^A lower score indicates a more positive response (inverted scores).

## Discussion

### Principal Findings

The study demonstrates the effectiveness of the self-help smartphone app *MCT & More* in students with depressive symptoms. As expected, the app led to a significant reduction in depressive symptoms and a significant increase in self-esteem in the intervention period of 4 weeks. In our study, a small effect size of *d*=0.26 (PHQ-9; PP sample) in reducing depressive symptoms was found, which is comparable with the effect size found in a meta-analysis of smartphone apps for depressive symptoms (*g*=0.22) [34] and is slightly higher than the effect size reported in a more recent meta-analysis in which the sample consisted of university students with depressive symptoms (*g*=0.18) [20]. In addition, a small to medium effect size of *d*=0.40 (RSE; PP sample) was found for the increase in self-esteem, which corresponds to the findings of a study on a self-help web-based intervention, which was mainly focused on the treatment of depressive symptoms [61]. Contrary to our expectations, the use of the self-help smartphone app did not lead to a significant increase in quality of life (WHOQOL-BREF). The WHOQOL-BREF defines quality of life as the individual perception of one’s own life situation [41]. It is possible that an improvement in the life situation only occurs after a longer period and would have been shown in follow-up examinations [61]. The quality of life was assessed using a single global item. It is possible that improvements would have been found on the subscales of the WHOQOL-BREF (eg, psychological quality of life and social relationships). In the intervention group and the wait-list control group, a significant reduction in depressive symptoms and a significant increase in self-esteem were observed after the intervention period of 4 weeks. Despite the improvements in the wait-list control group, significant group differences were found (significantly higher improvement in the intervention group). The improvement in the wait-list control group may be because of changes in external circumstances, the use of other services, or spontaneous remissions [62]. As the survey took place during the COVID-19 pandemic, depressive symptoms among students may have been more severe than usual [63]. In addition, the quality of life may have been reduced [64].

### Adherence and Acceptance

Most participants used the self-help smartphone app regularly (91/129, 75.8%) at least once a week; self-assessment) and completed the study (263/400, 65.8%). Participants who completed the postassessment expected a more positive treatment outcome at the baseline assessment than the participants who dropped out of the study (t_398_=−2.12; *P*=.04). This result was also found by Mira et al [36] and underlines the relevance of the expected treatment outcome at the beginning of the intervention for participants’ study adherence. The reduction in symptoms (PHQ-9) did not correlate with the frequency of use of the self-help smartphone app. Other researchers who investigated the relationship between frequency of use and symptom reduction also concluded that using apps with a medium frequency only leads to a little additional benefit than using apps with low frequency [65]. It should be considered that the assumption of a linear relationship between frequency of use and symptom reduction might be too simplistic and that further variables need to be evaluated to better understand the relationship. A possible explanation may also be that, because the intervention was not linear or sequential, participants may have been more likely to use only the parts of the intervention they needed. For some, a low dose may have been sufficient for symptom improvement, whereas other users may have required to use the program more often.

### Attitudes and Expectations

The students’ overall expectation of treatment outcomes and their attitude toward internet- and mobile-based interventions was moderate. Almost half of the students (192/400, 48%) did not expect any improvement from the app, and about one-third of the students (140/400, 35%) had a negative attitude toward internet- and mobile-based interventions. These findings are consistent with the results of another German study, in which 41% of the respondents (patients with depressive symptoms in primary care) indicated a low acceptance of internet- and mobile-based interventions for depression [66]. However, other studies conducted in Germany found a more positive attitude toward internet- and mobile-based interventions for depressive symptoms (total scale: mean 55.86, baseline total scale intervention group: mean 56.13, and baseline total scale control group: mean 55.59) [67,68]. It is possible that the attitude toward internet- and mobile-based interventions was lower in this study (mean 50.09; higher scores indicate a more positive attitude) because of the young age of the sample. In Germany, younger people report a more negative attitude toward internet- and mobile-based interventions than older people [69]. The attitude could possibly be improved by showing information videos that address potential barriers of acceptance (eg, low expectations regarding efficacy and worries about data security) before use [66]. Furthermore, the more positive the attitude toward internet- and mobile-based interventions and the more positive the expectations of the treatment outcome, the more frequently the self-help smartphone app was used, which was also found in other studies [70,71].

Most of the students trusted the therapeutic efficacy (328/400, 82%), which seems contradictory at first, as about half of the participants (192/400, 48%) did not expect any improvement. It is possible that the participants were generally convinced of the efficacy of internet- and mobile-based interventions (APOI queries a general attitude) but did not expect any improvement for themselves (PATHEV refers to their own symptomatology). Individuals with depressive symptoms often do not believe their symptoms will improve because they often express feelings of hopelessness [72].

The moderation analysis revealed that participants in the intervention group who indicated a higher expectation of treatment outcome (PATHEV total score) and more hope (PATHEV scale hope) achieved a higher reduction in depressive symptoms (PHQ-9) than those in the wait-list control group. The results of the linear regression showed that the attitude toward internet- and mobile-based interventions did not allow a prediction of the effectiveness. In addition, participants who were more worried that the app would not help them (PATHEV, item 5) showed less improvement in symptoms (PHQ-9). These findings are in contrast with the results of Lüdtke et al [37], who found no impact (moderation analysis) of expected treatment outcome (University of Rhode Island Change Assessment) on symptom reduction. This could be because of the use of different measurements. However, Schröder et al [68] found that participants with a more positive attitude (APOI) at the beginning of an internet intervention experienced a stronger reduction in symptoms than participants with a more negative attitude. Another recent study on the effectiveness of a self-help smartphone app for depression showed that the expected treatment outcome was a predictor of symptom reduction [36].

### Subjective Appraisal

The majority of the participants were satisfied with the quality of the self-help smartphone app (77/119, 64.7%) and the extent of help (66/119, 55.5%). Another study evaluating a cognitive behavioral therapy–based self-help web-based program among students with depressive symptoms also found a moderate overall satisfaction (study by Santucci et al [73]: total satisfaction score (ZUF-8): mean 21.70 (SD 5.20) and total satisfaction score (ZUF-8) of this study: mean 20.28 (SD 5.36)). In a pilot study of the self-help smartphone app *MCT & More*, the participants reported a higher overall satisfaction (eg, 88.5% of the participants were satisfied with the quality of the self-help smartphone app [37]). This could be because of the different average ages of the samples (pilot study: 43 years and this study: 23 years). Older age is associated with greater intervention effects [74] and more positive attitudes toward internet-based interventions [69], which may lead to higher satisfaction.

### Strength and Limitations

No psychiatric diagnosis was required to participate in the study, and participation was possible even with mild depressive symptoms, which led to a heterogeneity of depression levels. This has the advantage that a wider range of individuals with a desire for treatment was reached (regardless of whether they fulfilled the criteria of a diagnosis). On the other hand, it has been shown that individuals with severe depressive symptoms benefit more from low-threshold psychological interventions than mildly depressed individuals [75]. In contrast, a meta-analysis showed that self-guided internet-based interventions are effective regardless of symptom severity [17]. Furthermore, treatment adherence was rather high (91/120, 75.8% used the app at least once a week), which allowed the potential of the app to be well exploited. As the study was conducted on the web, the data collected were based on self-assessments of the participants. Therefore, it could not be eliminated that socially desirable or dishonest statements were made that could have distorted the results. In addition, despite the integrated control questions on studying, it could not be completely prevented that individuals who were not enrolled at a German university also took part in the study. An unambiguous verification of the student status (eg, via enrolment certificates) was not possible because of data privacy reasons. There was no structural equality between the sample and the general population (students in Germany) regarding gender and subject groups [13,76]. The higher proportion of women could be because women are more often affected by depression than men (women: 10.8% and men: 7.6%) and that this difference is particularly evident in young adulthood [77,78]. Furthermore, the study showed baseline differences regarding some comorbid self-reported diagnoses and the evaluation of previous therapy experiences. Nevertheless, randomization was largely considered successful. Follow-up investigations were not possible because of the time frame of the study. For this reason, no conclusions can be drawn regarding the medium- or long-term effects of the self-help smartphone app.

### Conclusions

The effectiveness of the self-help smartphone app *MCT & More* was demonstrated in students with depressive symptoms, although the overall outcome expectation and attitude toward internet- and mobile-based interventions were only moderate. Despite the improvements in the wait-list control group, significant group differences were found. The use of the app led to a significantly higher reduction in depressive symptoms (*d*=0.26) and a significantly higher increase in self-esteem (*d*=0.40). The expected treatment outcome and the attitude toward internet- and mobile-based interventions were correlated with the frequency of use. The more positive the attitude and the more positive the result expectation, the more frequently the self-help smartphone app was used. Participants who indicated a higher expectation of treatment outcome and more hope achieved a higher reduction in depressive symptoms. In addition, participants who were more worried that the app would not help them and participants who stated a higher reduced or excessive need to eat showed less improvement in symptoms.

Future studies should investigate further variables (with respect to personal characteristics and app features) that positively influence the effectiveness of identifying ways of increasing efficacy. To make self-help smartphone apps as target group–specific as possible, further subgroups should be identified for which a particularly high or low effectiveness is shown. In addition, follow-up studies are required to determine the long-term effects. It should be investigated how attitudes toward internet- and mobile-based interventions and the expected treatment outcome can be improved to establish effective self-help smartphone apps as low-threshold offers at universities and to promote treatment adherence. The self-help smartphone app could be used regularly at German universities as a low-threshold program to enhance students’ health.

## Appendix

Multimedia Appendix 1 CONSORT-eHEALTH checklist (V 1.6.1).

## Abbreviations

ANCOVA: analysis of covariance
APOI: Attitude Toward Psychological Online Interventions
CC: complete-case
CONSORT: Consolidated Standards of Reporting Trials
INEP: Inventory for Assessing Negative Effects of Psychotherapy
ITT: intention-to-treat
MCT: metacognitive training
PATHEV: Patient Questionnaire on Therapy Expectation and Evaluation
PHQ-9: Patient Health Questionnaire-9
PP: per-protocol
RSE: Rosenberg Self-esteem Scale
WHOQOL-BREF: World Health Organization Quality of Life-abbreviated version
ZUF-8: Fragebogen zur Patientenzufriedenheit (German version of the Client Satisfaction Questionnaire-8)
