# Correction: A Mobile-Based Intervention to Increase Self-esteem in Students with Depressive Symptoms: Randomized Controlled Trial

**DOI:** 10.2196/39448

**Published:** 2022-05-20

**Authors:** Alina Bruhns, Thies Lüdtke, Steffen Moritz, Lara Bücker

**Affiliations:** 1 University Medical Center Hamburg-Eppendorf (UKE) Hamburg Germany

In “A Mobile-Based Intervention to Increase Self-esteem in Students with Depressive Symptoms: Randomized Controlled Trial” (JMIR Mhealth Uhealth 2021;9(7):e26498), the authors noted the following errors.

In the originally published article, the effect size for self-esteem was incorrectly reported as *d*=0.77. The correct value should be *d*=0.40. Therefore, in relation to this value, the phrase “medium to large effect” should be corrected to “small to medium effect.” The effect sizes of all other result parameters were converted correctly.

Furthermore, in the originally published article, commas separating degrees of freedom in the *F* values were missing (eg, *F*_1,222_ was erroneously presented as *F*_1222_).

Pertaining to these two errors, the following corections were made in the article.

1. In “Abstract” (Results), the sentence “Per-protocol (PP), complete-case, and intention-to-treat analyses showed a significantly higher reduction in depressive symptoms (PP: *F*_1222_=3.98; *P*=.047; *d*=0.26) and a significantly higher increase in self-esteem (PP: *F*_1220_=8.79; *P*=.003; *d*=0.77) in the intervention group than in the wait-list control group” has been corrected to “Per-protocol (PP), complete-case, and intention-to-treat analyses showed a significantly higher reduction in depressive symptoms (PP: *F*_1,222_=3.98; *P*=.047; *d*=0.26) and a significantly higher increase in self-esteem (PP: *F*_1,220_=8.79; *P*=.003; *d*=0.40) in the intervention group than in the wait-list control group.”

2. In “Results” (Between-Group Differences), the phrase “The analyses resulted in a medium to large effect size for the increase in self-esteem (η_p_²=0.038; *d*=0.77) in the PP sample across time” has been corrected to “The analyses resulted in a small to medium effect size for the increase in self-esteem (η_p_²=0.038; *d*=0.40) in the PP sample across time.”

3. In “Discussion” (Principal Findings), the phrase “In addition, a medium to large effect size of *d*=0.77 (RSE; PP sample) was found for the increase in self-esteem,…” has been corrected to “In addition, a small to medium effect size of *d*=0.40 (RSE; PP sample) was found for the increase in self-esteem,….” 

4. In “Discussion” (Conclusion), the sentence “The use of the app led to a significantly higher reduction in depressive symptoms (*d*=0.26) and a significantly higher increase in self-esteem (*d*=0.77)” has been corrected to “The use of the app led to a significantly higher reduction in depressive symptoms (*d*=0.26) and a significantly higher increase in self-esteem (*d*=0.40).”

5. In “Abstract” (Results), “*F*_1222_” has been corrected to “*F*_1,222_” and “*F*_1220_” has been corrected to “*F*_1,220_.”

6. In “Results” (Between-Group Differences), “*F*_1398_” has been corrected to “*F*_1,398_,” “*F*_1223_” to “*F*_1,223_,” “*F*_1261_” to “*F*_1,261_,” “*F*_1221_” to “*F*_1,221_,” and “*F*_1259_” to“*F*_1,259_.”

7. In Table 3, “(1398)” has been corrected to “(1,398),” “(1222)” to “(1,222),” “(1220)” to “(1,220),” “(1223)” to “(1,223),” “(1261)” to “(1,261),” and “(1259)” to “(1,259).”

8. In “Results” (Attitude and Expectation), “*F*_1275_” has been corrected to “*F*_1,275_.”

The correction will appear in the online version of the paper on the JMIR Publications website on May 20, 2022, together with the publication of this correction notice. Because this was made after submission to PubMed, PubMed Central, and other full-text repositories, the corrected article has also been resubmitted to those repositories.

